# Can We Build an Evidence Base on the Impact of Systems Thinking for Wicked Problems? Comment on "What Can Policy-Makers Get Out of Systems Thinking? Policy Partners’ Experiences of a Systems-Focused Research Collaboration in Preventive Health"

**DOI:** 10.34172/ijhpm.2020.194

**Published:** 2020-10-14

**Authors:** Diane T. Finegood

**Affiliations:** Morris J. Wosk Centre for Dialogue, Simon Fraser University, Vancouver, BC, Canada.

**Keywords:** Systems Thinking, Complex Adaptive Systems, Evidence Base, Decision-Making, Research Impact

## Abstract

The published literature on the application of systems thinking to influence policies and programs has grown in recent years. The original article by Haynes et al and the subsequent commentaries have focused on the upstream connection between capacity building for systems thinking and systems informed decision-making. This commentary explores the downstream connection between systems-informed decision-making and broader impacts on the health system, the health of the population and other economic and social benefits. Storytelling, systems-based syntheses and systems intervention principles are explored as approaches to strengthen the evidence base. For systems thinking to gain broader acceptance and application to complex health-related challenges, we need more of an evidence base demonstrating impact.

## Upstream Impacts of Systems Thinking

The language and logic of systems thinking has slowly been gaining traction in healthcare and public health over the last couple of decades. Much has been written about the need for systems thinking when tackling complex or ‘wicked’ problems like obesity and chronic disease, but as Haynes et al point out the application of systems thinking to tackle complex policy problems can be challenging because the literature is “amorphous and often highly theoretical.”^1^ As example, there are many different definitions of systems thinking. For clarity and appropriateness, this paper uses the definition provided by The Australian Prevention Partnership Centre (TAPPC), the collaborative program of work studied by Haynes et al.^1^ TAPPC defines systems thinking as a “way to make sense of a complex system that gives attention to exploring the relationships, boundaries and perspectives in a system. It is a mental framework that helps us to become better problem solvers.”^2^ This paper focuses on the challenge of building an evidence base for systems thinking as a ‘mental framework.’

Helpfully, Haynes and colleagues add some empirical data to the published literature in their original article on the experience of policy-makers involved in projects with TAPPC, a national partnership of researchers, policy-makers and practitioners with an explicit commitment to co-producing practical systems-informed research, tools, resources and methods for tackling chronic disease prevention. Their work attempts to draw on a broad range of academic disciplines and apply systems methods to map challenges, identify leverage points and test intervention options.^1^ The study by Haynes et al set out to understand what policy-makers are getting out of this systems-infused collaboration with TAPPC.^1^ The authors found that their policy partners perceived that systems thinking has merit and through collaboration and capacity building these policy-makers can put systems thinking into action. They also presented some evidence that the application of systems thinking as part of a research collaboration can result in discernable impacts on the policy process.

These results can be described using the Canadian Academy of Health Sciences framework for measuring the return on investment in health research.^3^ Figure summarizes the more detailed framework which illustrates many influences on each of the components, including established practices, infrastructure, resources, traditions, political dynamics and technical limitations.^3^

**Figure:**
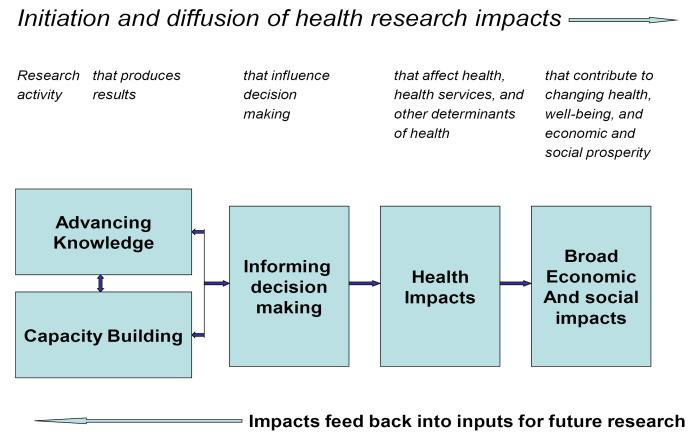
Framework for Measuring the Impact of Health Research. Adapted from the full model developed by an assessment panel for the Canadian Academy of Health Sciences.^3^

In this context, Haynes et al demonstrates a strong link between TAPPC’s effort to build capacity for systems thinking through projects that advance knowledge, and a shift towards systems-informed decision-making.^2^ The group of respondents who were engaged with systems thinking could identify concrete impacts on their work including development of practical methodologies for policy design, scaling up, implementation and evaluation. They were also able to articulate new prevention narratives. What remains unknown is whether these applications of systems thinking had downstream impacts on health, economic and/or social well-being.

Many of those who have already commented on this paper also focused on different aspects of capacity building including receptivity, commitment and co-production.^4-8^ Boswell et al suggests the lack of buy in by senior policy actors remains a persistent obstacle.^4^ Sturmberg also focuses on the clash between hierarchical bureaucracies and their leaders, and the need to consider real-world systems as a whole.^5^ Nyström et al suggests an important approach to addressing this clash is to focus on developing shared mental models.^6^ Khan argues the barriers to implementation in the policy sphere including capability, opportunity and motivation indicate that multiple approaches are required for capacity building.^7^ Holmes thinks the language of systems thinking might be a barrier, since many policy actors perceive they are already systems thinkers, but do not know the jargon of systems thinking.^8^ She suggests focusing on co-production as an important means of capacity building for systems-informed decision-making.

While these commentaries help us consider multiple challenges and opportunities to strengthen the links between our investments in capacity building for systems thinking and its impact on decision-making, none of them addresses the next connection between systems-informed decision-making and the evidence that this contributes to more effective programs and practices and better outcomes. Only Lamont pushes us along the impact framework by focusing on the tension policy-makers feel between embracing complexity and the need for hard evidence and stories of impact.^9^ Lamont reminds us that policy-makers live in a world where accountability and impact are deeply embedded in the culture and notes how these clash with a systems thinking frame. Policy-makers want toolkits with proven interventions and best practices,^9^ but as Snowden points out “best practices” are only possible for simple problems, whereas when challenges are complex practices are “emergent.”^10^

## What Does Evidence of the Impact of Systems Thinking Look Like?

Lamont suggests evidence can come in the form of stories of impact, given that stories are a form that allows for nuance and are important tools for moving knowledge to action.^9^ But it is hard to use stories to construct an evidence base especially in the absence of an appropriate framework.^11^ As Greenhalgh and Fahy demonstrated when the dominant framework is the linear logic model, the impacts of system thinking do not emerge.^12^ These authors did a manual content analysis of 162 impact narratives submitted to public health, health services and primary care section of the 2014 Research Excellence Framework. They found that the majority of the case studies described quantitative research (most commonly trials and systematic reviews) and depicted a direct, linear link between research and impact. Qualitative and participatory research designs were rare, and only one case study described a co-production model of impact.^12^

Another approach to providing evidence of the impact of systems thinking was developed by Leykum and colleagues.^13^ In a systematic review of organizational interventions to improve care of people with type II diabetes they scored interventions according to the number and type of characteristics of complex adaptive systems that were used in the intervention and examined this against the effectiveness of the intervention. The authors found that the number of complex adaptive systems characteristics present in the intervention was strongly associated with intervention effectiveness. Both interconnections between participants and co-evolution were independently associated with effectiveness.^13^ Leykum and colleagues’ approach to synthesis has been applied and adapted by others suggesting that characteristics of complexity could serve as the basis for a useful framework.

Quinn Patton introduced principles-focused evaluation as a more intervention-oriented approach to navigating and supporting change in complex environments.^14^ Quinn Patton argues principles can be guiding, useful, inspiring, developmental and evaluable. As an example, this approach has been used to guide the work of and also evaluate efforts to overcome youth homelessness.^14^ In the evaluation of this initiative all participating organizations were found to be adhering to the co-developed principles and the principles “were effective (even essential) in helping them make progress out of homelessness.”^14^ That the principles developed by collaborating organizations were found to have impact suggests that a focus on principles may offer a path towards systems-informed interventions that also enable the development of an evidence base. Could a general set of GUIDEing principles be developed for the application of systems thinking? If a set of principles forms a mental framework they could give rise to new methods and their applications. If interventions are based on a common set of principles, their evaluations could form an evidence base for impact.

Frameworks are powerful tools to advance methods and their application. The evidence-based medicine pyramid has dominated health sciences and public health for more than 30 years. As called for in our Lancet commentary, we need a new framework or mental model for evidence that embraces complexity.^11^ Although many models and frameworks for complexity exist (eg, Cynefin,^10^ Stacy, iceberg and intervention level frameworks^15^), none has emerged as a key driver of new methods and their application in the published literature. Whether a focused effort to synthesize currently popular frameworks could produce a common framework that helps to grow the evidence base for systems thinking remains to be determined, but the illustrative examples provided here suggest such a framework is possible.

## Ethical issues

Not applicable.

## Competing interests

The author has served as a member of the International Scientific Advisory Board of The Australian Prevention Partnership Centre since 2014. The author is also serving as the co-chair of an Expert Advisory Group leading a UK-Canada collaboration on systems thinking and methods for complex health challenges. The project secretariat is provided by a collaboration between the Canadian Academy of Health Sciences and the UK Academy of Medical Sciences with funding from The Wellcome Trust and The Health Foundation.

## Author’s contribution

DTF is the single author of the paper.
